# Synaptic Protection in the Brain of *Wld^S^* Mice Occurs Independently of Age but Is Sensitive to Gene-Dose

**DOI:** 10.1371/journal.pone.0015108

**Published:** 2010-11-29

**Authors:** Ann K. Wright, Thomas M. Wishart, Cali A. Ingham, Thomas H. Gillingwater

**Affiliations:** 1 Euan MacDonald Centre for Motor Neurone Disease Research, University of Edinburgh, Edinburgh, United Kingdom; 2 Centre for Integrative Physiology, University of Edinburgh, Edinburgh, United Kingdom; 3 Royal (Dick) School of Veterinary Studies, University of Edinburgh, Edinburgh, United Kingdom; National Institutes of Health, United States of America

## Abstract

**Background:**

Disruption of synaptic connectivity is a significant early event in many neurodegenerative conditions affecting the aging CNS, including Alzheimer's disease and Parkinson's disease. Therapeutic approaches that protect synapses from degeneration in the aging brain offer the potential to slow or halt the progression of such conditions. A range of animal models expressing the slow Wallerian Degeneration (*Wld^S^*) gene show robust neuroprotection of synapses and axons from a wide variety of traumatic and genetic neurodegenerative stimuli in both the central and peripheral nervous systems, raising that possibility that *Wld^S^* may be useful as a neuroprotective agent in diseases with synaptic pathology. However, previous studies of neuromuscular junctions revealed significant negative effects of increasing age and positive effects of gene-dose on *Wld^S^*-mediated synaptic protection in the peripheral nervous system, raising doubts as to whether *Wld^S^* is capable of directly conferring synapse protection in the aging brain.

**Methodology/Principal Findings:**

We examined the influence of age and gene-dose on synaptic protection in the brain of mice expressing the *Wld^S^* gene using an established cortical lesion model to induce synaptic degeneration in the striatum. Synaptic protection was found to be sensitive to *Wld^S^* gene-dose, with heterozygous *Wld^S^* mice showing approximately half the level of protection observed in homozygous *Wld^S^* mice. Increasing age had no influence on levels of synaptic protection. In contrast to previous findings in the periphery, synapses in the brain of old *Wld^S^* mice were just as strongly protected as those in young mice.

**Conclusions/Significance:**

Our study demonstrates that *Wld^S^*-mediated synaptic protection in the CNS occurs independently of age, but is sensitive to gene dose. This suggests that the *Wld^S^* gene, and in particular its downstream endogenous effector pathways, may be potentially useful therapeutic agents for conferring synaptic protection in the aging brain.

## Introduction

Synaptic connections are an early pathological target in many neurodegenerative conditions, ranging from Alzheimer's disease, prion diseases and Batten disease through to childhood and adult forms of motor neuron disease [1]–[8]. Therapies directly addressing synaptic degeneration are therefore actively being sought for a wide range of neurological disorders. One promising avenue of research in this area focuses on understanding the neuroprotective properties of the slow Wallerian degeneration (*Wld^S^*) gene and its potential use as a novel synaptoprotective agent.

The *Wld^S^* gene confers strong neuroprotection upon synaptic and axonal compartments of neurons following injury in both the peripheral and central nervous systems [9], [10], but has no direct effect on neuronal soma [11]. These neuroprotective properties significantly modify disease onset and/or progression in animal models of chemically-induced Parkinson's disease [12]–[13], demyelinating neuropathies [14], some forms of motor neuron disease [15] and global cerebral ischemia [16], highlighting the potential use of *Wld^S^* and/or its downstream mediators to generate novel therapeutic approaches for the treatment of neurological disorders. Importantly, it has also been demonstrated that the *Wld^S^* gene can be used to confer robust neuroprotection *in vivo* using delivery methods including gene therapy [17], [18], and that systemic expression the *Wld^S^* gene has no overt detrimental effects on other non-neuronal systems, tissues or organs [19].

The chimeric *Wld^S^* gene resulted from a spontaneous mutation in the C57BL/6 line of mice, causing a tandem triplication in the distal region of chromosome 4 [20]. Mice carrying the *Wld^S^* gene are otherwise indistinguishable from their C57BL/6J strain mates in genotyping of more than 50 microsatellite markers and restriction fragment length polymorphisms [21]–[23]. The triplicated region of chromosome 4 contains two copies of a fusion gene comprising the N70 terminal amino acids of Ube4b and the entire coding region of Nmnat1 (C Terminal 285 amino acids), linked by 18 amino acids from the 5′ untranslated region of Nmnat1 [20]–[21], [24]. The chimeric portion of the triplication (i.e. the N-70 Ube4b/Nmnat1 C-303 chimera) is sufficient to confer the full *Wld^S^* phenotype in mice, rats and drosophila [25]–[27], although its mechanism of action remains controversial [18], [28]–[32].

One potential caveat regarding the usefulness of the *Wld^S^* phenotype for protecting synapses in the brain concerns an age-dependent aspect to *Wld^S^*–mediated synaptic protection previously revealed in the peripheral nervous system (PNS) [33]. This is of particular importance as many neurodegenerative diseases affecting the brain are associated with an aging population [34]–[35]. In studies of axotomy-induced synaptic degeneration at the neuromuscular junction *Wld^S^* mice older than 4 months began to lose their synaptoprotective phenotype, with no synaptic protection observed at all in mice older than 7 months [33]. By contrast, axonal protection conferred by *Wld^S^* in the PNS was shown to be age-independent but highly sensitive to gene dose [25], [33], [36]. Similarly, synaptic protection in the PNS is known to be highly sensitive to *Wld^S^* gene dose [37]. Whether the synaptoprotective phenotype observed in the CNS of young *Wld^S^* mice [10], [31] is also sensitive to age and/or gene-dose has yet to be determined.

Here, we have undertaken a detailed study of the effects of gene-dose and age on synaptic protection in the brain of *Wld^S^* mice *in vivo*. We show that synaptic protection medicated by *Wld^S^* in the CNS occurs independently of age, but is sensitive to *Wld^S^* expression levels.

## Results

### Synaptic protection in the striatum of *Wld^S^* mice is sensitive to gene-dose

We have previously demonstrated robust protection of synapses in the striatum following lesion to the cortico-striatal pathway in young (∼2 mth old) *Wld^S^* mice [10]. We used the same model system to assess the sensitivity of CNS synapses to *Wld^S^* gene-dose by quantifying synaptic pathology in the striatum of 2 mth old wild-type (−/−), heterozygous *Wld^S^* (+/−) and homozygous *Wld^S^* (+/+) mice following a cortical lesion. Experimental mice were generated from heterozygous *Wld^S^* (+/−) breeding pairs in order to allow within litter comparisons.

**Figure 1:**
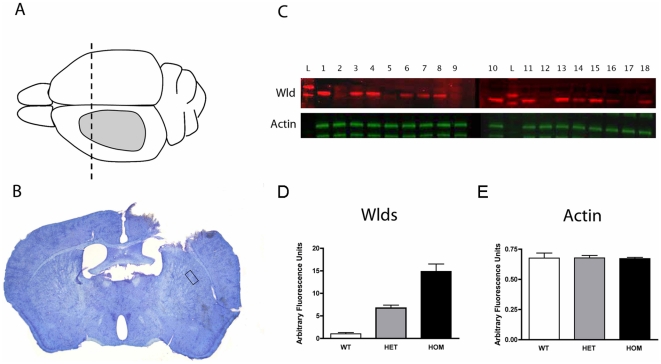
Cortical lesion model for initiating synaptic degeneration in the striatum of wild-type, heterozygous *Wld^S^* and homozygous *Wld^S^* mice. A/B – Schematic diagram of the mouse brain viewed from above (A), showing the extent of cortical lesion produced (grey area). The dotted line in panel A represents the level of brain shown in coronal section in panel B (note the lesion to the left cortex). The box in panel B shows the region of striatum selected for ultrastructural experiments. C – Quantitative fluorescent western blotting of protein extracted from tail tips was used to confirm the genotype of experimental mice generated from heterozygous *Wld^S^* (+/−) breeding colonies. Example blots show Wld^S^ protein levels (red; labelled with the Wld-18 antibody specific for Wld^S^ protein) and levels of actin loading control (green) in tail tips from 18 mice. 2 membranes are shown side by side with randomly arranged samples from individual mice numbered 1–18. A molecular weight marker is also shown (L). Lanes numbered 2,5,9,12 and 17 show wild-type mice (no Wld^S^ protein present), lanes 6,7,8,14,16 and 18 show heterozygous *Wld^S^* mice (intermediate levels of Wld^S^ protein present), and lanes 1,3,4,10,11,13 and 15 show homozygous *Wld^S^* mice (high levels of Wld^S^ protein present). D/E – Bar charts (mean±SEM) showing quantification of fluorescent western blots (see methods) shown in panel C (pooled to give a mean value for each genotype), confirming that heterozygous *Wld^S^* mice had approximately half the expression levels of Wld^S^ protein observed in homozygous *Wld^S^* mice (D), whilst levels of actin loading control remained constant (E) across mice of all genotypes. N = 5 wild-type mice, 6 heterozygous *Wld^S^* mice & 7 homozygous *Wld^S^* mice.

Synaptic degeneration was induced in the striatum by performing a lesion in the ipsilateral neocortex of wild-type, heterozygous *Wld^S^* and homozygous *Wld^S^* mice using aspiration under general anaesthesia (Figure 1A–B) [10]. Mice were allowed to recover and were maintained for 3 days. A 3 day post-lesion time-point was selected for examination as a result of previous experimental data showing that the largest difference in synaptic responses to injury between wild-type and homozygous *Wld^S^* mice occurred at 3 days post-lesion [10]. All mice recovered fully following surgery with no discernable difference between the genotypes. Following sacrifice and perfusion fixation for electron microscopy (see methods) the extent of the cortical lesion was mapped in all mice to ensure comparability (Figure 1B). The region of striatum selected for analyses of synaptic degeneration was between 0.7 and 1.06 mm posterior to bregma, directly under the corpus callosum at the level of the ventral border of the lateral ventricle (Figure 1A–B) [38]. Wld^S^ protein levels in the brain were determined using quantitative fluorescent western blotting. As expected, wild-type mice showed no expression but heterozygous mice had roughly half the levels of Wld^S^ protein observed in homozygous mice (Figure 1C).

**Figure 2:**
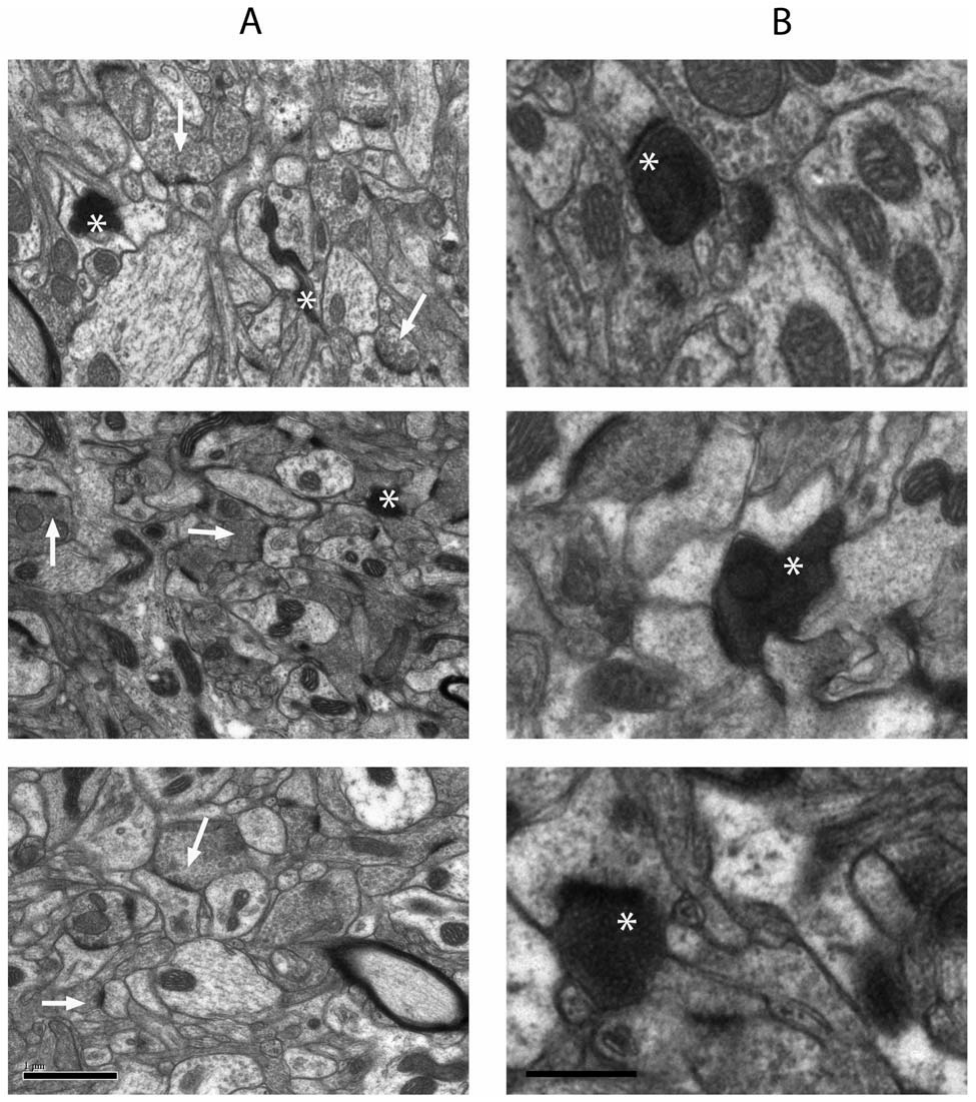
Widespread synaptic degeneration in the striatum of young (2 month old) wild-type, but not heterozygous *Wld^S^* or homozygous *Wld^S^* mice, 3 days after cortical lesion. Representative electron micrographs of striatal synapses at low power (A) and higher power (B) from a young wild-type mouse (top panel), heterozygous *Wld^S^* mouse (middle panel) and homozygous *Wld^S^* mouse (bottom panel). Asterisks indicate degenerating synaptic profiles (identified principally by their electron dense cytoplasm) and arrows indicate healthy (i.e. non-degenerating) synaptic profiles. Degenerating synapses were readily identified in wild-type mice, occasionally observed in heterozygous *Wld^S^* mice and rarely observed in homozygous *Wld^S^* mice. However, the morphological appearance of degenerating synapses was indistinguishable between the different genotypes, suggesting that synapses in mice expressing the *Wld^S^* gene ultimately degenerate by the same mechanism as in wild-type mice, albeit after a delay. Scale bars; A = 1 µm, B = 0.5 µm.

Qualitative ultrastructural analysis of synapses in the striatum of wild-type mice 3 days after cortical lesion revealed a mixture of normal synaptic profiles (identifiable as vesicle-laden boutons separated by a synaptic cleft from a post-synaptic density; Figure 2) as well as degenerating synapses, identified by their dark, electron-dense cytoplasm and/or disrupted synaptic organelles (Figure 2) [10], [39]–[40]. These characteristics of degenerating synapses are widely accepted to be the best early morphological indicators of synaptic degeneration [10], [39], [40] and we did not identify any obvious intermediate states preceding these morphological alterations in our material. As in our previous study [10], virtually all degenerating nerve terminals formed part of an asymmetrical synapse, consistent with the glutamatergic nature of corticostriatal projections (Figure 2). Qualitative assessment of heterozygous *Wld^S^* mice showed that degenerating synapses were present, but not in the same numbers observed in wild-type animals. Degenerating synapses were also present, but rare, in homozygous *Wld^S^* mice. Degeneration of post-synaptic striatal neurons was not observed in any of the mice examined, in agreement with previous studies suggesting that striatal neurons do not degenerate following denervation [40]–[41].

**Figure 3:**
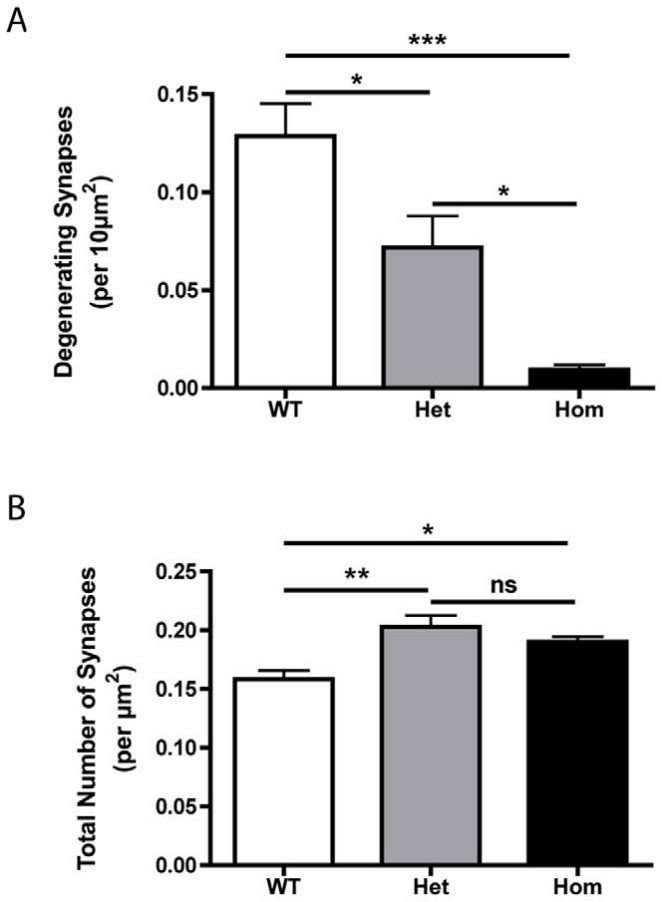
Quantitative analysis of synaptic degeneration confirmed a dose-dependent protection of striatal synapses in *Wld^S^* mice 3 days after cortical lesion. A – Bar chart (mean±SEM) showing the number of degenerating synapses in the striatum of wild-type (WT), heterozygous *Wld^S^* (Het) and homozygous *Wld^S^* (Hom) mice 3 days after cortical lesion (***P<0.001, *P<0.05, ANOVA with Tukey's post-hoc test; N = 4 wild-type mice, 3 heterozygous *Wld^S^*, 3 homozygous *Wld^S^*). B – Bar chart showing the total number of synapses remaining in the striatum of wild-type (WT), heterozygous *Wld^S^* (Het) and homozygous *Wld^S^* (Hom) mice 3 days after cortical lesion (**P<0.01, *P<0.05, nsP>0.05, ANOVA with Tukey's post-hoc test; N = 4 wild-type mice, 3 heterozygous *Wld^S^*, 3 homozygous *Wld^S^*).

Quantitative analyses of synaptic degeneration confirmed our qualitative observations of dose-dependent CNS synaptic protection in *Wld^S^* mice (Figure 3). Randomly-generated micrographs of striatal tissue (see methods) were independently assessed by 3 experienced investigators. The results reported in Figure 3 are a mean value from the 3 independent counts. All investigators were blind to the genotype of the images being analysed. More than 23,000 individual synapses from 10 mice (4 wild-type, 3 heterozygous *Wld^S^* and 3 homozygous *Wld^S^)* were examined and classified as either healthy or degenerating. At 3 days after cortical lesion there were significantly more degenerating synaptic profiles in the striatum of heterozygous *Wld^S^* mice compared to homozygous *Wld^S^* mice (Figure 3A; P<0.05 ANOVA with Tukey's post-hoc test). There were also significantly more degenerating synaptic profiles in the striatum of wild-type mice compared to heterozygous *Wld^S^* mice (Figure 3A; P<0.05). Similarly, quantification of total (i.e. healthy and degenerating) synaptic densities revealed a significant reduction in wild-type mice compared to homozygous and heterozygous *Wld^S^* mice (Figure 3B). This demonstrated that the progression from degeneration to complete loss of individual synapses had only commenced in the wild-type mice, with the delay in degeneration resulting in a retention of synapses in *Wld^S^-*expressing mice.

**Figure 4:**
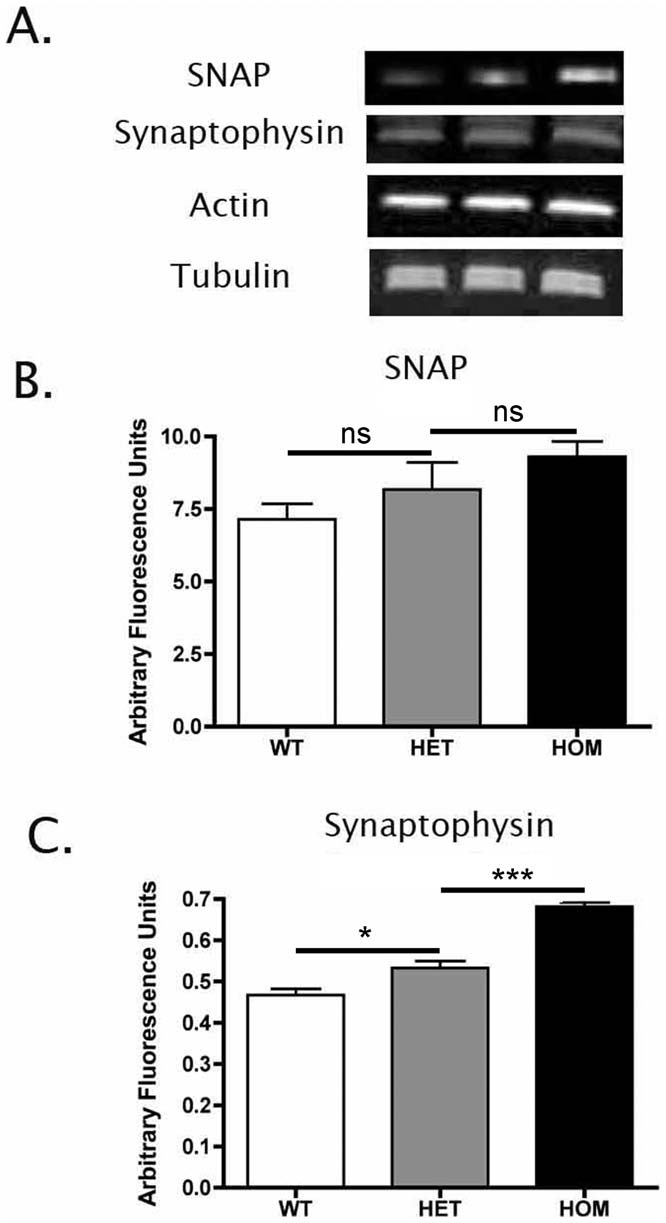
Quantitative western blotting for synaptic proteins confirmed dose-dependent protection of striatal synapses in *Wld^S^* mice 3 days after cortical lesion. A – Representative bands from western blots showing expression levels of two major synaptic proteins (SNAP and synaptophysin) as well as two loading controls (actin and tubulin) in the striatum of wild-type, heterozygous *Wld^S^* and homozygous *Wld^S^* mice 3 days after cortical lesion. Note lower levels of synaptic markers in heterozygous *Wld^S^* mice compared to homozygous *Wld^S^* mice and lower still levels in wild-type mice, indicative of a loss of synapses. B/C – Bar charts (mean±SEM) showing relative expression levels of SNAP (B) and synaptophysin (C) in the striatum of wild-type, heterozygous *Wld^S^* and homozygous *Wld^S^* mice 3 days after cortical lesion (ns = not significant, *P<0.05, ***P<0.001; ANOVA with Tukey's post-hoc test; N = 3 mice per genotype). Levels of actin and tubulin remained constant between samples (data not shown but see Panel A).

To validate our ultrastructural findings and confirm the dose-dependency of synaptic protection in the brains of *Wld^S^* mice, we examined synaptic degeneration in freshly prepared lesioned tissue samples using quantitative fluorescent western blotting for synaptic proteins (Figure 4). Expression levels of beta-SNAP were reduced in the striatum of wild-type mice after cortical lesion compared to homozygous *Wld^S^* mice, with heterozygous *Wld^S^* mice showing an intermediate level of loss (Figure 4A,B). However, despite showing similar trends to our ultrastructural data, the differences did not reach statistical significance due to relatively large variability between samples (Figure 4B). As beta-SNAP is a relatively soluble protein and is not as enriched at synapses as other synaptic markers, we repeated the experiment using antibodies against synaptophysin and observed significant differences in levels between homozygous *Wld^S^*, heterozygous *Wld^S^*, and wild-type mice (Figure 4A,C). These results suggest that synaptophysin is a more sensitive marker for synaptic pathology in the brain than beta-SNAP.

Taken together, these data reveal that synaptic protection in the CNS of *Wld^S^* mice is sensitive to gene-dose. These findings also confirm that the cortical lesion model is sensitive enough to detect subtle changes in levels of synaptic degeneration and protection *in vivo* using both ultrastructural and molecular approaches.

### Synaptic protection in the striatum of *Wld^S^* mice is not diminished with increasing age

**Figure 5:**
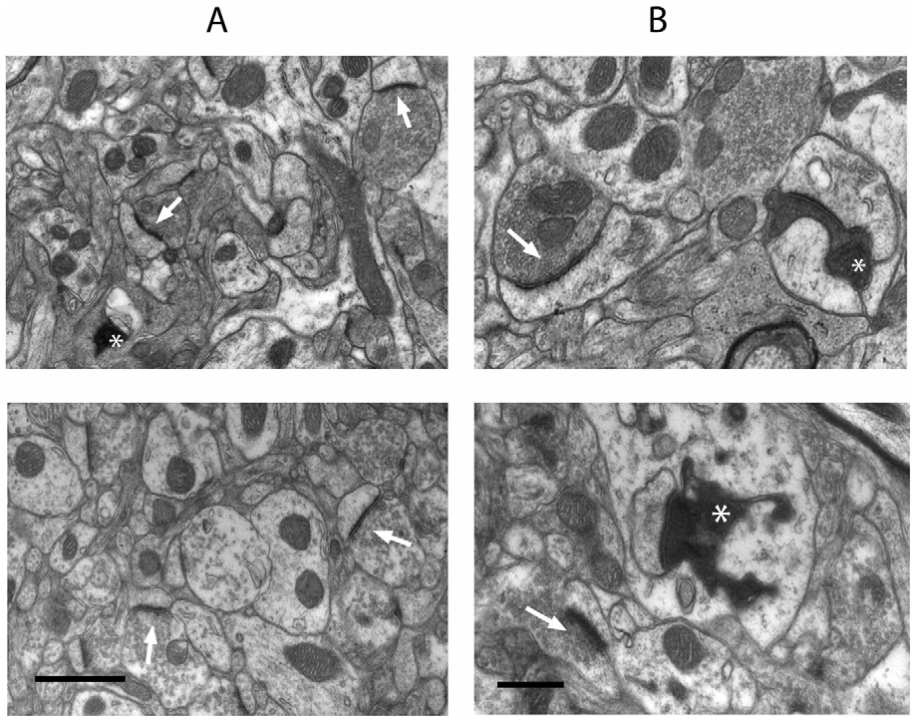
Synaptic protection in the striatum remains robust in old (∼12 month old) homozygous *Wld^S^* mice after cortical lesion. Representative electron micrographs of striatal synapses at low power (A) and higher power (B) from an old wild-type mouse (top panel) and old homozygous *Wld^S^* mouse (bottom panel) 3 days after cortical lesion. Asterisks indicate degenerating synaptic profiles (identified principally by their electron dense cytoplasm) and arrows indicate healthy (i.e. non-degenerating) synaptic profiles. Degenerating synapses were readily identified in wild-type mice but rarely observed in homozygous *Wld^S^* mice. As in young mice (see Figure 2), the morphological appearance of degenerating synapses was indistinguishable between the different genotypes. Scale bars; A = 1 µm, B = 0.5 µm.

To test whether age influences the level of synaptic protection conferred by *Wld^S^* in the CNS, we performed cortical lesions in old wild-type and *Wld^S^* mice (aged ∼12 months) and examined synaptic pathology at 3 and 5 days post-surgery. Levels of Wld^S^ protein in the brains of old *Wld^S^* mice were at least as high as those found in young (2 month old) *Wld^S^* mice (data not shown) [33]. Qualitative ultrastructural analysis of synapses in the striatum of old wild-type mice at both 3 and 5 days after cortical lesion revealed a mixture of normal and degenerating synapses (Figure 5). Degenerating synapses in old wild-type mice were morphologically indistinguishable from those previously observed in young mice (c.f. Figure 2 with Figure 5). In contrast, degenerating synapses were rarely observed in old *Wld^S^* mice at either 3 or 5 days post-lesion (Figure 5).

**Figure 6:**
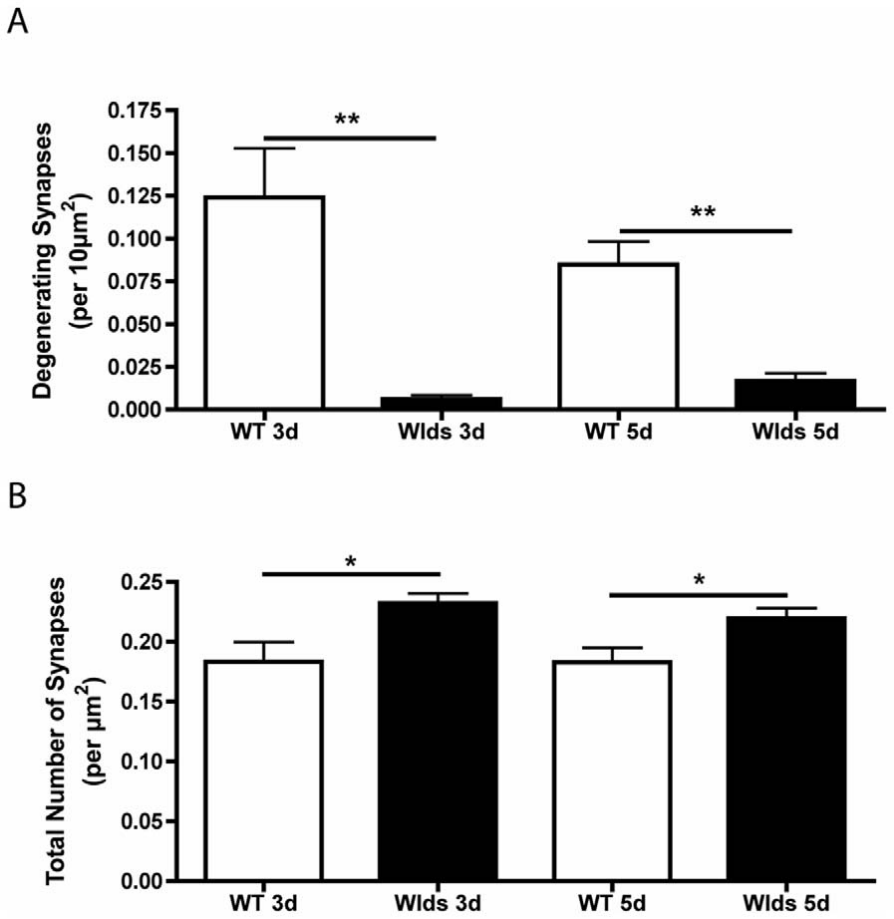
Quantitative analysis of synaptic degeneration confirmed robust protection of striatal synapses in old (∼12 mth) *Wld^S^* mice 3 and 5 days after cortical lesion. A – Bar chart (mean±SEM) showing the number of degenerating synapses in the striatum of wild-type (WT) and homozygous *Wld^S^* (Wlds) mice at 3 and 5 days after cortical lesion (**P<0.01, ANOVA with Tukey's post-hoc test; N = 4 wild-type mice per time-point, 3 homozygous *Wld^S^* mice per time-point). B – Bar chart showing the total number of synapses remaining in the striatum of wild-type and homozygous *Wld^S^* mice at 3 and 5 days after cortical lesion (*P<0.05, nsP>0.05, ANOVA with Tukey's post-hoc test; N = 4 wild-type mice per time-point, 3 homozygous *Wld^S^* mice per time-point).

Quantitative analyses, using the same methodology described above for dose-dependency experiments, confirmed that increasing age had no influence on synaptic protection in the CNS of *Wld^S^* mice (Figure 6). At both 3 and 5 days after cortical lesion there were significantly more degenerating synaptic profiles in the striatum of wild-type mice compared to homozygous *Wld^S^* mice (Figure 6A; P<0.01 ANOVA with Tukey's post-hoc test). Similarly, there were significantly fewer synapses remaining in wild-type striatum compared to homozygous *Wld^S^* tissue at both 3 and 5 days after cortical lesion (Figure 6; P<0.05 ANOVA with Tukey's post-hoc test). Thus, synaptic protection in the CNS of *Wld^S^* mice was not modified by age and was as robust in old CNS synapses as in young CNS synapses.

## Discussion

In this study we examined the influence of gene-dose and increasing age on synaptic protection in the brain of mice expressing the *Wld^S^* gene. We demonstrate that synaptic protection in the striatum was sensitive to *Wld^S^* gene-dose following cortical lesion, with heterozygous *Wld^S^* mice showing approximately half the level of protection observed in homozygous *Wld^S^* mice. In contrast, we show that increasing age had no influence on levels of synaptic protection in the brain, revealing different synaptoprotective phenotypes in the CNS and PNS of old (>7 mth) *Wld^S^* mice. Taken together, these findings suggest that *Wld^S^*-mediated neuroprotection is likely to be of potential therapeutic benefit for conferring synaptic protection in the aging brain. Importantly, our finding that synaptic protection in the brain is sensitive to *Wld^S^* gene-dose suggests that delivery of higher levels of *Wld^S^* to neurons could provide robust long-lasting synaptic protection, even in the aging nervous system.

Our finding that synaptic protection in the brain of old *Wld^S^* mice occurred just as robustly as in young (∼2 mth) *Wld^S^* mice is in stark contrast to events occurring at synapses in the mouse PNS. It is well established that full *Wld^S^*-mediated protection of neuromuscular synapses is only present in mice up to 4 months of age, with mice of 7 months or older reverting to wild-type synaptic degeneration characteristics [33]. The reasons for this difference remain unclear. One possibility is that synaptic degeneration occurs via subtly distinct mechanisms in the CNS and PNS. This hypothesis is supported by data from current and previous experiments [10] which demonstrated that brain synapses degenerate by the same cellular pathways in wild-type and *Wld^S^* mice (albeit delayed in the latter), whereas neuromuscular synapses in *Wld^S^* mice degenerate via a withdrawal-like mechanism morphologically and mechanistically distinct from the classical Wallerian degeneration process that occurs in wild-type mice [33]; [42]–[43]. It is possible, therefore, that the pathways regulating synaptic degeneration in the PNS have age-dependent characteristics that do not impact on pathways regulating synaptic degeneration in the brain.

A recent study from Beirowski and colleagues demonstrated that the efficacy of *Wld^S^*–mediated protection is regulated by protein levels in the cytoplasm and/or non-nuclear organelles, rather than protein levels in the nucleus [44]. This raises the possibility that the strong, age-independent synaptic protection we observed in the brain was occurring due to neurons in the brain having consistently higher non-nuclear levels of Wld^S^ protein expression than motor neurons in the periphery. Alternatively, data from transgenic rats expressing the *Wld^S^* gene suggest that the age-dependent characteristics of neuroprotection at peripheral synapses are dependent on the presence of long distal nerve stumps, particularly in older animals [26]. This dependency on nerve stump length might not be an important factor for CNS neurons. Future experiments addressing issues of non-nuclear Wld^S^ protein expression and nerve stump length for synaptic protection in the brain are therefore warranted.

Regardless of the differences observed between the CNS and PNS, the current study raises the exciting possibility that the *Wld^S^* gene, and in particular its downstream endogenous effector pathways, could potentially be useful therapeutic agents for conferring synaptic protection in the aging brain and central nervous system. Systemic delivery of the *Wld^S^* gene targeting neurones in the brain is possible using gene therapy approaches [17], [18], and *Wld^S^* gene expression is safe and well-tolerated by other body systems and organs [19]. However, a more interesting and realistic therapeutic approach would be to target endogenous cellular pathways that are themselves targeted by *Wld^S^* in neurones. Recent breakthroughs in our understanding of the mechanism of action of *Wld^S^* have highlighted several potential effector pathways and sub-cellular targets that may be suitable as therapeutic targets [28], including; Nmnat-dependent pathways [18], [45]–[46], mitochondria [31], [47]–[48], VCP-dependent pathways [49], [50], and cell cycle/cell stress pathways [32], [51]. The current study suggests that manipulating one or several of these pathways or targets, in a similar manner and extent to that generated by the *Wld^S^* gene *in vivo*, should confer synaptic protection in the aging brain. Downstream endogenous mediators of synaptic protection conferred by the *Wld^S^* gene are therefore attractive targets for designing synaptoprotective therapeutic strategies for age-related neurodegenerative diseases affecting the brain, including Alzheimer's disease and Parkinson's disease.

## Materials and Methods

### Ethics statement

All animal experiments were approved by a University of Edinburgh internal ethics committee and were performed under license by the UK Home Office (project license number 60/3891).

### Mice

Natural mutant homozygous C57Bl6/Wld^S^ (*Wld^S^*) mice and C57Bl6 (wild-type) mice aged 2 months were obtained from Harlan Olac Laboratories (Bicester, UK) and housed within the animal care facilities in Edinburgh. A breeding program was undertaken to obtain mice heterozygous for the *Wld^S^* gene. These heterozygous mice were then cross-bred to obtain litters containing mice homozygous and heterozygous for the *Wld^S^* gene as well as wild-type littermates, facilitating within litter comparisons. Young mice were used for experiments at 6-10 weeks of age. A cohort of mice were also maintained in animal care facilities in Edinburgh for the aging experiments. Mice were genotyped using either qRT-PCR [52] or quantitative fluorescent western blotting (see below) [19].

### Surgery

All operations were performed under licence from the UK Home Office. General anaesthesia was induced using a mixture of isopentane and oxygen, before securing the head in a Kopf stereotaxic frame. Fur overlying the cranial vault was shaved with scissors before making an incision through the skin at the midline. Four holes were drilled on the left side of skull; 1) in the midline at bregma, 2) in line with the first but at the level of lambda, 3) further caudal on the lateral side just above the temporalis muscle, 4) anterolateral in line with the first and third holes. The skull was cut in lines connecting all holes except the most caudal border, and then reflected. A suction pipette was used to remove all visible cortex under a dissecting microscope, down to the level of the corpus callosum, before replacing the skull-flap. The lesion site was filled with gel foam (Ethicon) before replacing the skull-flap. Overlying skin was then sutured and the mouse placed on a heated blanket until recovered fully from the anaesthetic. Mice were maintained in standard animal house conditions and were checked daily for any signs of distress or discomfort.

### Electron microscopy

Three days after surgery, anaesthetised mice were killed by perfusion fixation with 0.1 M phosphate buffer containing 4% paraformaldehyde and 2.5% glutaraldehyde, before removing the brain and immersing in fixative for a further 12–24 hrs. Brains were washed in 0.1 M phosphate buffer before cutting free-floating 70 µm thick coronal sections on a vibratome. The region of striatum used for analysis of synaptic pathology was located between 0.70 and 1.06 mm posterior to Bregma, directly under the corpus callosum at the level of the ventral border of the lateral ventricle. Vibratome sections containing this region were post-fixed in 1% osmium tetroxide for 45 minutes and dehydrated through an ascending series of ethanol solutions (including dehydration for 40 mins in 70% alcohol containing 1% uranyl acetate) and propylene oxide. Sections were embedded on glass slides in Durcupan resin. Regions of striatum (∼1 mm×1 mm) to be used for quantitative assessment were cut out from a randomly selected section using a scalpel and glued onto a resin block for ultrathin sectioning. Ultrathin sections (∼50–70 nm) were cut and collected on formvar-coated grids (Agar Scientific, UK), stained for 10 mins with lead citrate, and then viewed using a Philips CM12 transmission electron microscope. Images were captured directly using a Gatan digital camera and were quantified using Image J software (version 1.35c).

Images were taken at random locations within each grid (e.g. starting at the top corner of the section before progressing across the section in a direction dictated by the loading position of the grid in the microscope) and quantified independently by 3 investigators, with each of the investigators blind to the genotype of the images being assessed. Average data from the three individual assessments were used for comparison between genotypes. Standard ultrastructural characteristics were used to define healthy and degenerating synapses. Normal synaptic terminals were located by the presence of vesicle-filled pre-synaptic boutons, separated from their post-synaptic target by a clear synaptic cleft. Degenerating synapses were identified by their dark, electron dense cytoplasm, often containing disrupted synaptic vesicles and mitochondria, consistent with previous ultrastructural studies of degenerating synapses [10], [39]–[40]. Numbers of degenerating and healthy synapses were counted in each electron micrograph. Of those synaptic profiles that crossed a border of the micrograph, only those that crossed the top and left hand borders of the image were included, with those crossing the bottom and lower borders being excluded.

### Western blotting

For genotyping mice, tail tips were removed from freshly sacrificed mice and assayed for the level of Wld^S^ protein expression. This was possible as Wld^S^ protein has previously been shown to be expressed in a wide range of tissues and organs, including muscle and skin of the tail [19]. Brains for quantification of synaptic proteins were rapidly removed and briefly chilled in ice cold ACSF (125 mM NaCl, 26 mM NaHCO3, 25 mM glucose, 2.5 mM KCl, 1.25 mM NaH2PO4, 1 mM CaCl2, 4 mM MgCl2) before striata were microdissected out.

Quantitative fluorescent western blotting was performed as previously described [53]–[55]. Briefly, membranes were incubated with primary antibodies as per manufacturers instructions (synaptophysin – Dako; β actin, β SNAP & βIII tubulin – Abcam). Wld-18 antibodies, selective only for the chimeric Wld^S^ protein [16], [19], [25], were a kind gift from Dr Michael Coleman (Babraham Institute, Cambridge). Odyssey secondary antibodies were added according to manufacturers instructions (Goat anti rabbit IRDye 680 or 800 and Goat anti mouse IRDye 680 or 800 – dependant on required comparisons). Blots were imaged using an Odyssey Infrared Imaging System (Li-COR Biosciences) at either 84 µm or 169 µm resolution.

### Statistical analysis

All data were collected into Microsoft Excel spreadsheets and analysed using GraphPad Prism software. All bar charts shown are mean ± SEM. Statistical significance was considered to be p<0.05 for all analyses. Individual statistical tests used are detailed in figure legends.
